# Do we miss rare adverse events induced by COVID-19 vaccination?

**DOI:** 10.3389/fmed.2022.933914

**Published:** 2022-10-10

**Authors:** Zeinab Mohseni Afshar, Ali Tavakoli Pirzaman, Jackson J. Liang, Akanksha Sharma, Marzieh Pirzadeh, Arefeh Babazadeh, Erfan Hashemi, Niloofar Deravi, Sadaf Abdi, Amirreza Allahgholipour, Rezvan Hosseinzadeh, Zahra Vaziri, Terence T. Sio, Mark J. M. Sullman, Mohammad Barary, Soheil Ebrahimpour

**Affiliations:** ^1^Clinical Research Development Center, Imam Reza Hospital, Kermanshah University of Medical Sciences, Kermanshah, Iran; ^2^Student Research Committee, Babol University of Medical Sciences, Babol, Iran; ^3^Division of Cardiovascular Medicine, Cardiac Arrhythmia Service, University of Michigan, Ann Arbor, MI, United States; ^4^Department of Neurology, Mayo Clinic, Scottsdale, AZ, United States; ^5^Infectious Diseases and Tropical Medicine Research Center, Health Research Institute, Babol University of Medical Sciences, Babol, Iran; ^6^Student Research Committee, School of Medicine, Shahid Beheshti University of Medical Sciences, Tehran, Iran; ^7^Student Research Committee, School of Nursing and Midwifery, Shahid Beheshti University of Medical Sciences, Tehran, Iran; ^8^Department of Radiation Oncology, Mayo Clinic, Phoenix, AZ, United States; ^9^Department of Social Sciences, University of Nicosia, Nicosia, Cyprus; ^10^Department of Life and Health Sciences, University of Nicosia, Nicosia, Cyprus; ^11^Student Research Committee, Virtual School of Medical Education and Management, Shahid Beheshti University of Medical Sciences, Tehran, Iran; ^12^Students' Scientific Research Center (SSRC), Tehran University of Medical Sciences, Tehran, Iran

**Keywords:** COVID-19, SARS-CoV-2, drug-related side effects and adverse reactions, vaccination, pandemic (COVID-19)

## Abstract

Although severe acute respiratory syndrome coronavirus 2 (SARS-CoV-2) infection has caused many complications, the invention of coronavirus disease 2019 (COVID-19) vaccines has also brought about several adverse events, from common side effects to unexpected and rare ones. Common vaccine-related adverse reactions manifest locally or systematically following any vaccine, including COVID-19 vaccines. Specific side effects, known as adverse events of particular interest (AESI), are unusual and need more evaluation. Here, we discuss some of the most critical rare adverse events of COVID-19 vaccines.

## Introduction

**Figure 1:**
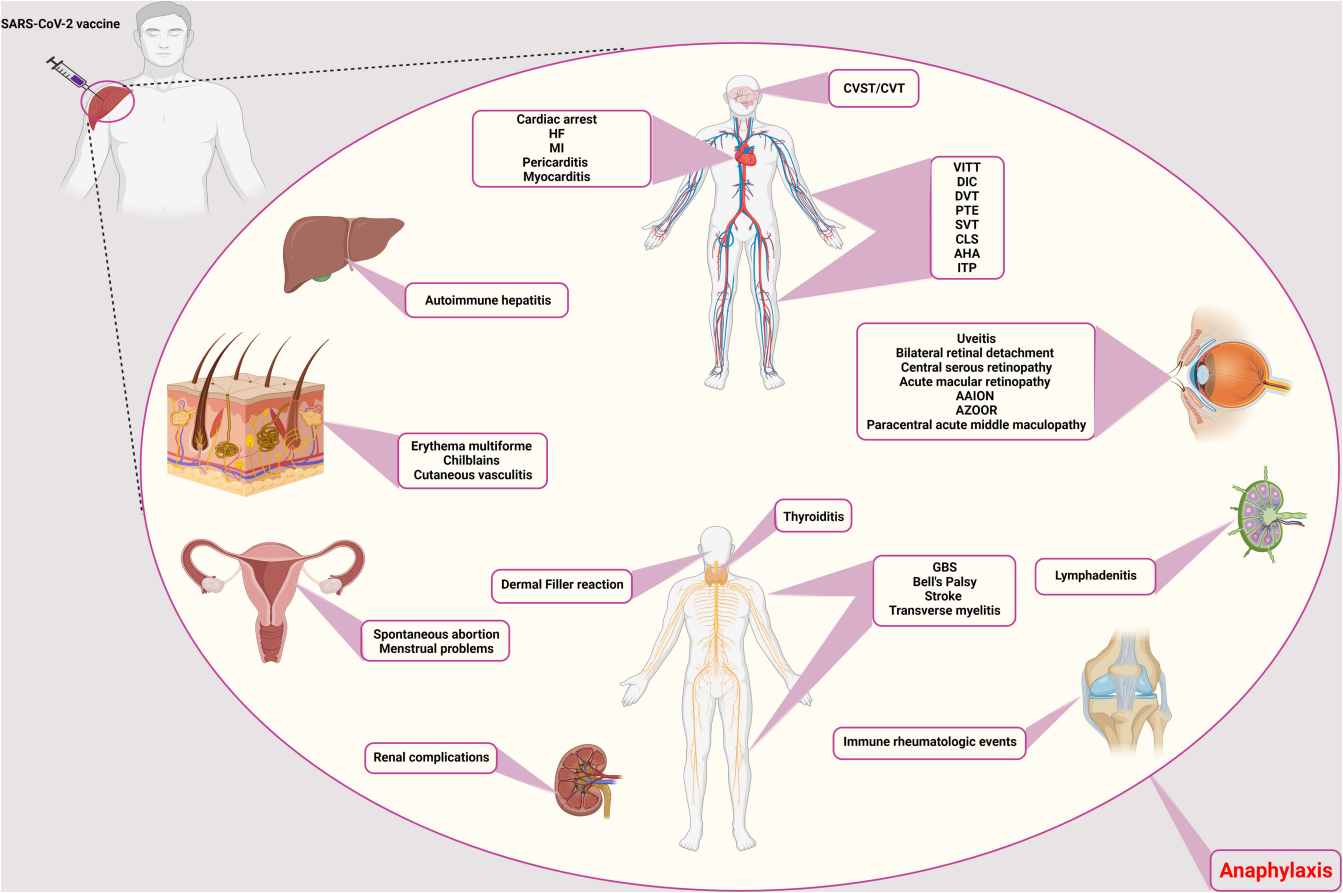
Rare adverse events of COVID-19 vaccination. The side effects of the COVID-19 vaccines are different and can affect different systems and tissues in the body. Vascular side effects have been seen in the brain, vascular system of the limbs, abdomen, and heart, including CVST/CVT, VITT, DIC, DVT, PTE, CLS, AHA, ITP, SVT, cardiac arrest, HF, MI, pericarditis, and myocarditis, respectively. Ocular involvement includes uveitis, bilateral retinal detachment, central serous retinopathy, acute macular retinopathy, AAION and AZOOR, and paracentral acute middle maculopathy. The thyroid gland can also cause thyroiditis. Neurological side effects such as GBS, Bell's palsy, stroke, and transverse myelitis have also been observed. It causes filler on the face. In addition to facial involvement, skin infections such as erythema multiforme, chilblains, and cutaneous vasculitis have also been reported. It causes autoimmune hepatitis in the liver and has caused many complications for the kidneys. Symptoms of immune rheumatologic events have also been observed in some patients. Lymphadenitis is one of the immune complications in the lymph nodes. In addition to the above, it also causes spontaneous abortion and menstrual problems in women. CVST/CVT, Cerebral venous sinus thrombosis/Cerebral venous thrombosis; VITT, Vaccine-induced immune thrombotic thrombocytopenia; DIC, Disseminated intravascular coagulation; DVT, Deep vein thrombosis; PTE, Pulmonary thromboendarterectomy; CLS, Capillary leak syndrome; AHA, Acquired hemophilia A; ITP, Immune thrombocytopenic purpura; SVT, Supraventricular tachycardia; HF, Heart failure; MI, Myocardial infarction; AAION, Arteritic anterior ischemic optic neuropathy; AZOOR, Acute zonal occult outer retinopathy; GBS, Guillain-Barré syndrome.

Although severe acute respiratory syndrome coronavirus 2 (SARS-CoV-2) infection has caused many complications, the invention of coronavirus disease 2019 (COVID-19) vaccines has also brought about several adverse events from common side effects to unexpected and rare ones (1). Common vaccine-related side adverse reactions are those manifested locally or systematically following any vaccine, including COVID-19 vaccines. Local reactions include erythema, tenderness, induration, and, rarely, abscess formation at the injection site. In contrast, systemic side effects include fever, chills, headache, cough, coryza, and rarely anaphylactic reactions (2). However, specific side effects, known as adverse events of special interest (AESI), could manifest as autoimmune diseases or involve various organs, such as renal, dermatologic, hematologic, lymphatics, ocular, gastrointestinal, cardiovascular, and neurologic systems, are unusual and need more evaluation (1). Some adverse events have never happened with other previously administered vaccines (Figure 1). Examples are those related to the hyperthrombotic condition induced by the COVID-19 vaccines that have been discussed in several articles (3). Some rare adverse events have been discussed among the reported side effects of COVID-19 vaccines. Table 1 encompasses the reported frequencies of these adverse events. First, their proposed pathophysiology is described, and the possible diagnostic approaches along with recommended treatment options are demonstrated. Finally, most of the following adverse events discussed in this paper are very uncommon. Since there is no formal evidence that vaccines are responsible in most cases, it has to be considered that establishing a cause-effect relationship is challenging and needs more investigation into the actual occurrence of the complication and the underlying pathophysiology.

**Table 1:** Frequency of rare vaccine-related and COVID-19-related complications.

**Complication**	**Frequency or the number of reported cases**	
	**Vaccine-related complication**	**COVID-19-related complication**
Acquired hemophilia A	18^a^	7^a^
Immune-mediated thrombocytopenia	1.13^b^	Not determined
Anaphylaxis	1.11^b^	Not determined
Capillary leak syndrome	4^a^	101
IgA vasculitis	19	13
Urticarial vasculitis	3^a^	6^a^
Cutaneous vasculitis	11^a^	7^a^
Arthritis	−0.8^b^	Not determined
IgA nephropathy	50^a^	12^a^
Thyroiditis	51	17
Dermal filler reaction	14	Not determined
Renal complications (acute kidney injury)	−4.6^b^	125.4^b^
Erythema multiforme	8	36
Disseminated intravascular coagulation	Not determined	3%^c^
Deep vein thrombosis	−1.1^b^	43.0^b^
Pulmonary thromboembolism	−1.5^b^	61.7^b^
Splanchnic vein thrombosis	Not determined	21
Lymphadenopathy	78.4^b^	Not determined
AAION and AZOOR	2^a^	2^a^
Acute macular neuro-retinopathy	19^a^	12^a^
Paracentral acute middle maculopathy	5^a^	14^a^
Central serous retinopathy/chorioretinopathy	11^a^	11^a^
Bilateral Retinal Detachment	4^a^	2^a^
Uveitis	1.0^b^	Not determined
Autoimmune hepatitis	32	Not determined
Myocardial infarction	0.8^b^	25.1^b^
Myocarditis	2.7^b^	11.0^b^
Pericarditis	1.0^b^	10.9^b^
Guillain-Barré syndrome	43	99
Stroke	−1.6^b^	Not determined
Bell's palsy	3.5^b^	Not determined
Transverse myelitis	31	40

^a^Number of reported cases bases on PubMed. ^b^Per 100,000 doses. ^c^The incidence of DIC in COVID-19 patients.

## Autoimmune complications

### Acquired hemophilia A

Acquired hemophilia A (AHA) is a rare autoimmune-hematologic disorder within the immune system that is aroused by a trigger to produce auto-antibodies against clotting factor VIII (FVIII inhibitor) (4). It is suggested to be associated with predisposing factors, such as autoimmune diseases, drugs, pregnancy, infections, and malignancy. AHA major presentations include bruise or ecchymosis in the skin, mainly in the extremities, but it may progress to muscles and mucosal layers. However, joint involvement is not included despite the hereditary type (5). Recently, few case reports have introduced SARS-CoV-2 mRNA vaccines as a possible trigger for AHA without other predicting conditions (6–8). There are also several reports on exhibiting AHA following SARS-CoV-2 infection (5). Presumed pathophysiology can be the antigenic mimicry of SARS-CoV-2 and FVIII, leading to uncontrollable degradation of FVIII. Autoimmune response may be conducted by various underlying causes such as particular genetic polymorphism and activation of previously existing autoimmune B/T cells (9). Laboratory confirming evidence includes prolonged activated partial thromboplastin time (aPTT), decreased level of FVIII, and elevated FVIII inhibitor (4). Mentioned cases diagnosed with vaccination caused AHA within days to weeks following receiving first/second doses of Moderna (mRNA1273) or Pfizer-BioNTech (BNT162b2) vaccines (5, 6, 8, 9). Cases were mainly discharged after a few days of treatment. AHA treatment methods consist of two major issues: (1) homeostasis normalization. First, it is vital to complete the coagulation cascade with recombinant activated factor VII (rFVIIa) and activated prothrombin complex concentrate (aPCC); and (2) immune suppressive therapy should be conducted by corticosteroids (mainly high dose prednisone), cyclophosphamide, and rituximab (in refractory cases) (4). Given the very low incidence of AHA following the COVID-19 vaccines, which might be a coincidental event (8), it can be challenging to define a cause-effect relation between AHA and vaccination, and it is not wise to limit the COVID-19 vaccination. Instead, it is better to continue investigations to clarify whether there is any cause-effect relation.

### Immunomediated thrombocytopenia

Immunomediated thrombocytopenia (ITP) is an autoimmune disorder in which platelets destroy by autoantibodies. Etiology primarily includes autoimmunity but it can also occur secondary to viral infections and even vaccination. In the past years, after the beginning of the COVID-19 vaccination, several ITP cases have been reported, mainly following mRNA vaccines (10). The adjusted RR for ITP, 0–27 days following ChAdOx1 vaccination, was 5.77 (estimated incidence: 1.13 events per 100,000 doses). Nevertheless, there was no association between BNT162b2 vaccine and ITP (11). In terms of *de novo* ITP, which happens within days, hypothesized pathophysiology encompasses molecular mimicry and cross-reaction of antibodies against vaccine components and platelet antigens (9). Albeit, in case of a flare-up of pre-existing ITP occurring in hours, the underlying mechanism amplifies prior immune response (9). It is worth noting that the measured incidence of secondary ITP after vaccination has been lower than expected ITP in the general population so far (12). However, as the exact differentiation of coincidental ITP and ITP related to vaccination is impossible, immediate treatment should be performed to prevent severe outcomes (12). Patients present with mild-to-severe symptoms due to the platelet count. In moderate thrombocytopenia, symptoms generally include petechiae, purpura, bruising, and mucosal bleeding (13). Platelet count under 5,000/μL is life-threatening with a high risk of intracranial hemorrhage (ICH) (13). Treatment typically consists of IV fluids, corticosteroids, IVIG, platelet transfusion, rituximab, and thrombopoietic agents (e.g., romiplostim, eltrombopag) (13). A better approach has been suggested to start the initial treatment with corticosteroids (e.g., dexamethasone, methylprednisone) and IVIG (13). Furthermore, if the platelet count did not rise, other agents could be added. Since rituximab responds in about 8 weeks and alters the body's reaction to the vaccine, it should be excluded from initial treatment (13). Although many studies have reported ITP as a possible complication of COVID-19 infection (14–21), fewer have reported ITP occurring following SARS-CoV-2 vaccines, and its risk is too less than the infection-induced ITP (22).

### Anaphylaxis

There are two types of adverse events following immunization (AEFI) after receiving vaccines; non-allergic reactions are normal expected immune responses, including pain in the injection site, nausea, fever, chill, and fatigue, while allergic reactions are caused by body hypersensitivity to vaccines' adjuvant (not an active ingredient) such as excipients. Allergic reactions may involve any body part and can exhibit mild-severe symptoms in different patients, including flush, pruritus, facial edema, tachycardia, laryngeal edema, and diarrhea (23). Anaphylaxis is perceived as an allergic AEFI with a very low incidence among vaccine receivers (1 in a million doses) and a higher rate in women, which is increasingly garnering attraction these days (24). Underlying pathophysiology has been proposed to be IgE-mediated type one hypersensitivity (25). However, there are a few other explanations as well. For example, complement activation pseudo-allergy (CARPA) is mainly instigated through C3a and C4a components without the involvement of IgE. Direct interaction of double-stranded RNA applied in vaccines and mast cells is also possible, but it is reported to be unlikely due to a lack of evidence in previous *in vitro* studies (26). For an IgE-mediated allergy, there should be prior contact with the allergen. To further decipher, polyethylene glycol (PEG) or Macrogol and polysorbate 80 have been reported as the most suspected component for vaccine-related anaphylaxis. PEG2000 has been utilized in SARS-CoV-2 mRNA vaccines as an excipient to facilitate mRNA delivery into cells. Note that it has a widespread application in medications and foods. Moreover, polysorbate 80 is a PEG-derived molecule with a lower weight recruited in many vaccines and medications (23). Their similar structures might lead to a cross-reactivity. It is worth noting that most of the reported cases had a prior allergy to foods, drugs, or vaccines (27).

Anaphylaxis mainly occurs in the first 15 min after vaccination. Symptoms mainly encompass sensation of throat closure, upper airway swelling, nausea-vomiting, tachycardia, difficulty breathing without wheezing or stridor, angioedema, hypotension, and dry cough (27). Diagnosis is primarily clinical and needs immediate action because it can be potentially fatal despite its very low incidence. Treatment includes urgent intra-muscular injection of epinephrine (0.01 mg/kg) and assessing airway, breathing, circulation, and mental status at the same time (23). Epinephrine injection repeats every 5–15 min if the symptoms stay resilient up to the maximum allowed dose of 0.5 mg in adults (0.3 mg in prepubertal children) (23). Antihistamine and glucocorticoids are second-line drugs mainly for skin and mucosal reactions. Note that PEG or polysorbate has been added to some of this medication which should not use as a treatment in patients with suspected allergy to these components (23). Post-vaccination anaphylaxis is among the severe adverse effects of COVID-19 vaccines. However, it has been reported as an infrequent complication (2.5–4.8 cases/million doses in adults) (28). Interestingly, mRNA-based vaccines have caused more cases of post-vaccination anaphylaxis (4.8 cases/million and 5.1 cases/million after BNT162b2 and Moderna vaccines, respectively) (29), which could be attributed to the application of PEG2000 in these type of vaccines. Since post-vaccination anaphylaxis is a far less common adverse effect, the definition of a cause-effect relationship can be challenging and needs future studies to be focused on this occasion.

### Capillary leak syndrome

Capillary leak syndrome (CLS) is the leakage of fluids in the vessels into the extravascular space. There are two types of classification for this disease: (1) idiopathic with unknown cause and secondary to underlying causes, (2) pulmonary CLS (PCLS), which mainly involves the lung and pleura, and systemic CLS (SCLS), which rarely consists of lungs edema (30, 31). Predisposing factors for secondary CLS include hematologic malignancies, medical treatment, and viral infections (32). In the last year, several case reports of developing pulmonary or systemic CLS following SARS-CoV-2 infection and vaccination (30, 33). Precise pathophysiology has yet needed to be elucidated. However, the assumed mechanism is dysregulation in inflammatory response leading to cytokine storm (i.e., viral sepsis) (31). High levels of pro-inflammatory agents and a hypoxic condition, pathogens' invasion, and immune cells' activity injure the epithelial-endothelial integrity causing permeability in the vascular wall and extravasation of exudative fluids (31). It has been demonstrated that hypoalbuminemia is a frequent finding in COVID-19 cases, representing the severity of injury to the epithelial-endothelial barrier (31). Both PCLS and SCLS have been reported after SARS-CoV-2 infection so far (30). However, CLS cases following COVID-19 vaccination had a previously diagnosed SCLS or suspicious history of SCLS symptoms (33, 34). SCLS generally leads to anasarca, hypotensive shock, hemoconcentration, hypoalbuminemia, monoclonal gammopathy, compartment syndrome, and multiple organ failure in more severe cases (33). It is worth noting that the diagnosis of SCLS is a diagnosis of exclusion (other causes of shock) (32). Lab data predominantly encompass hypoalbuminemia and increased level of creatinine, lactate dehydrogenase (LDH), creatine kinase (CK), and aspartate aminotransferase (AST) (18). The interval between vaccine administration and appearance of symptoms was about 1–2 days (18). For treatment, it has been suggested that the underlying conditions and ongoing damages should be managed. For example, albumin administration may even exacerbate the edema because vascular permeability is present continuously (18). Therefore, agents preserving epithelial-endothelial integrity can be beneficial, including solnatide, FX06, and Bβ15-42. Vasopressors (e.g., norepinephrine, vasopressin, and epinephrine), antibiotics, volume replacement, high dose of corticosteroid, IVIG (1 g/kg) are also pivotal. In the case of compartment syndrome, fasciotomy, and in the case of acute renal injury, renal replacement therapy (RRT) may be needed (18). oreover, prophylaxis with IVIG has been proposed as prevention in patients with prior SCLS who undergo vaccination (18).

Until today, several studies have reported SCLS as a complication of both COVID-19 and its vaccines (33–39). According to the Medicines and Healthcare products Regulatory Agency (MHRA) in the UK, SCLS has occurred in 13 cases among over 49 million shots of the ChAdOx1-S vaccine, and it has been advised not to use this vaccine in patients with a previous history of capillary leak syndrome (40). Since there is no clear evidence about the exact incidence of post-vaccination SCLS, at this moment, it is far too difficult to conclude that there is a cause-effect connection between SCLS and COVID-19 vaccination.

### IgA vasculitis and leukocytoclastic vasculitis (hypersensitivity vasculitis)

Another worrisome issue observed following COVID-19 vaccination is the probability of new-onset emergence or exacerbation of pre-existing autoimmune diseases (41). There have been rare reports of IgA vasculitis reactivation, previously known as Henoch-Schönlein Purpura, following the COVID-19 vaccination (42–44). IgA vasculitis causes skin, joints, intestines, and kidneys' small blood vessels to inflate and bleed. The pathogenesis underlying IgA vasculitis is yet to be known completely. However, the role of genetic, environmental factors, vaccines, malignancies, and infections have been reported (45). The most common feature of the disease is the presence of purplish, especially on the buttocks and lower legs. Elevated CRP, ESR, Urea, Creatinine, serum amyloid A levels, IgM, IgA, and anti-spike IgG could suggest IgA vasculitis associated with COVID-19 vaccination. Methylprednisolone, Deflazacort, and Paracetamol were prescribed in the mentioned cases (42, 45). Similarly, cases of leukocytoclastic vasculitis were reported following COVID-19 mRNA-based vaccines. Histopathological evaluations and direct immunofluorescence analysis helped make the diagnosis of leukocytoclastic vasculitis. Prednisolone taper was prescribed for the mentioned cases (46–48).

In the case of 18F-FDG PET/CT imaging, only very few studies have utilized this method in the cases of vasculitis following COVID-19 infection (49, 50). In the study of Sollini et al. 18F-FDG PET/CT findings suggested that post-COVID-19 vasculitis could be considered a cause of prolonged symptoms (fatigue, dyspnea, and chest pain) following COVID-19 recovery (50). Nevertheless, the existing evidence is not enough to advise 18F-FDG PET/CT imaging for all cases, and future investigations must be conducted to determine whether it is rational to use this method in both post-COVID-19 and post-vaccination vasculitis.

### Urticarial vasculitis

Urticarial vasculitis (UV) has been reported as a complication both after COVID-19 infection and the vaccination (51, 52). It is an inflammatory skin disorder manifested by the presence of urticarial rashes lasting more than a day and healing with hyperpigmentation. COVID-19 vaccine-induced UV is characterized by elevated red rashes on the skin, which are itchy. Elevated CRP and histopathological evaluations also help the diagnosis of the disease. In this case, oral indomethacin, levocetirizine tablet, and topical calamine lotion could be prescribed (52). Although some etiologies have been reported for UV, the idiopathic form is still the most common form of UV (53), and given that there are very few reports about both post-COVID-19 infection and post-COVID-19 vaccination UV, it is still far too soon to report a cause-effect relation.

### Cutaneous vasculitis

Cutaneous vasculitis is an inflammatory disease that affects dermal blood vessel walls. The skin is usually involved. Cutaneous vasculitis can reflect a cutaneous component of systemic vasculitis, a skin-dominant or skin-limited expression or variant of systemic vasculitis, or be a single-organ vasculitis *per se*. The diagnosis is often based on physical examination and skin biopsy (54). The main histological finding is a recently reported peculiar post-COVID-19 vaccination maculopapular rash characterized by lymphocytic vasculitis. The rash responded to systemic antihistamine and local steroid therapy (55).

The exact incidence of post-COVID-19 vaccination cutaneous vasculitis has not been determined until now, and there are only a few case reports presenting this complication (56, 57). Interestingly, a recent case report by Uh et al. has presented five cases of cutaneous small-vessel vasculitis following the ChAdOx1-S COVID-19 vaccine (58). After all, like all the other rare adverse effects following COVID-19 vaccination, it faces us with a challenging question of whether there are any cause-effect relationships or not.

### Rheumatoid arthritis and reactive arthritis

There have been reports of a rheumatoid arthritis flare-up following the COVID-19 vaccination. Moreover, rheumatoid arthritis exacerbation has also been reported after the COVID-19 vaccination (59), which had been previously observed following tetanus, rubella, hepatitis B, and influenza vaccines (60). The mechanism underlying this flare-up could be possibly attributed to the molecular mimicry or non-specific adjuvant effect. Elevated ESR and CRP levels with abnormal ultrasound evaluation of the swollen limb and arthrocentesis were suggestive of the flare-up of rheumatoid arthritis in these cases. Intra-articular steroids could be prescribed in these cases (61). Besides, a case of reactive arthritis has been reported following the SARS-CoV-2 vaccine in a 23-year-old woman treated with intra-articular betamethasone (59, 62). Despite the cases above, rheumatoid arthritis flare-up following the COVID-19 infection has also been reported (63).

### IgA nephropathy

IgA nephropathy has been similarly reported following COVID-19 mRNA vaccines and the infection itself (64, 65). However, like most of the adverse events discussed in this paper, it is very uncommon, and establishing a cause-effect relationship is difficult and needs more investigation. IgA nephropathy is a complex immune disorder with IgA deposition in the mesangial layer. The “multi-hit hypothesis” is almost the most accepted underlying pathophysiology. An increased level of galactose deficient IgA1 (Gd-IgA1) is needed for the progression of IgA nephropathy and IgA vasculitis-induced nephritis. Then, IgG immunoglobulins, targeting these Gd-IgA1 antibodies, appear and form some immune complexes with the Gd-IgA1, leading to an inflammatory state. Nevertheless, the exact mechanism by which COVID-19 leads to the generation of Gd-IgA1 immunoglobulins is not entirely understood and needs more investigation (66). However, there are some possibilities. For example, since COVID-19 is a mucosal infection, it could lead to Gd-IgA1 formation through increasing IL-6 production, resulting in impaired IgA1 glycosylation/galactosylation (67).

Furthermore, in these cases, the development of IgA nephropathy could be speculated due to an elevated response of immune cells in the germinal center, leading to massive antibody production and increased production of pathogenic IgA similar to immunization with the influenza vaccine. The cases had gross hematuria. Urine analysis, Kidney ultrasound, evaluation of immunoglobulin A levels, and kidney biopsy were suggestive of IgA nephropathy in the mentioned cases. Losartan and methylprednisolone were prescribed in these cases (68–70).

### Thyroiditis

Subacute thyroiditis, also known as De Quervain's thyroiditis, might be another rare adverse event with an immunological source associated with the COVID-19 vaccination (71–75). There are also a few reports about this complication after COVID-19 infection (76–78). It is a self-limited thyroid inflammation for weeks to months that commonly happens following a viral upper respiratory tract infection (79, 80). This possible post-vaccine effect has been presented with new-onset thyroid dysfunction in recently vaccinated individuals and appears to be of female predominance. The relationship between subacute thyroiditis and the type of COVID-19 vaccine is unclear as it has occurred following various vaccine platforms, including Sinovac, AstraZeneca, Bharat, Moderna, and Pfizer/BioNTech. This phenomenon was previously reported following H1N1, seasonal influenza, and hepatitis B vaccines (81–84). Vaccines' adjuvants are supposed to be responsible for these reactions by stimulating immunogenic cross-reactivity, causing autoimmune/inflammatory syndrome induced by adjuvants (ASIA syndrome) (75, 85). After vaccination, subacute thyroiditis can develop due to ASIA syndrome, including COVID-19 vaccines (85). The development of ASIA syndrome could be attributed to molecular mimicry, polyclonal activation of B cells, and immunological imbalance of the host (75). In addition to ASIA syndrome, the interaction between SARS-CoV-2 spike protein with angiotensin-converting enzyme 2 (ACE2) receptor, which is wildly expressed on thyroid cells, is another mechanism associated with thyroiditis induction in vector-based vaccines such as AstraZeneca (72). Clinical manifestations associated with thyroiditis include pharyngitis, moderate fever, diffuse myalgia, and cervical pain that radiates to the jaw and ears. Subacute thyroiditis is often associated with negative anti-thyroglobulin and anti-thyroid peroxidase antibodies (80).

In the mentioned cases, suppressed thyroid-stimulating hormone (TSH) levels accompanied by elevated triiodothyronine (T3) and thyroxine (T4), increased levels of inflammatory markers (ESR, CRP), ultrasound findings, negative thyroid antibodies helped in diagnosing subacute thyroiditis. Methylprednisolone, propranolol, and ibuprofen were prescribed in these cases (75, 80, 86). In this relation, cases of Grave's disease have also been reported following receiving the SARS-CoV-2 vaccine with elevated anti-thyroid antibodies (87).

### Dermal filler reaction

Hyaluronic acid (HA) is a natural polysaccharide that has been widely utilized in cosmetics (88). Reaction to HA fillers is rare and typically self-limited. So far, triggers such as infections (e.g., flu-like illnesses) and vaccinations (e.g., influenza vaccine) have been reported to exhibit filler reactions. Furthermore, several cases have been identified following anti-SARS-CoV-2 mRNA vaccines recently. Two cases with dermal fillers had developed swelling in lips and face in the third phase of the Moderna vaccine trial (89). There are two possible explanations for its pathophysiology. The first assumption is that the action of non-active components in vaccines cross-reacting with filler's molecules and provokes an immune response (90). The second hypothesis supports the alleviation of ACE2 conversion by mRNA vaccines, leading to pro-inflammatory ACE2 in the skin and inflammation. Reactions are type four hypersensitivity or delayed immune reactions mediated by T lymphocytes (90). Patients generally present with flu-like symptoms and swelling of the filler region days after vaccination. Tenderness, swelling, erythema, and nodules can be seen in the examination (90). Since most of the lesions have been resolved spontaneously, observation and follow-up are the first approach. However, in case of not improving nodules with pain, tenderness, or erythema, intervention is necessary (90). Antibiotics, including tetracycline and macrolides, should be administered for 3–5 days. If the nodule is non-inflammatory, hyaluronidase with or without intralesional steroids can be utilized to resolve it (90). Moreover, drainage of the nodule should be considered if the mass fluctuates. Interestingly, in some trials, low doses of ACE inhibitors have been used for 3–5 days, which significantly improved the reactions (90).

### Systemic sclerosis

Systemic sclerosis (SSc) is a rare chronic inflammatory connective tissue disease that can cause vascular abnormalities, organ involvement, and skin fibrosis (91). Pulmonary fibrosis and pulmonary hypertension (PAH) are observed in most of these patients due to increased serum levels of TNF-α (92). Etanercept and infliximab are among the drugs that can reduce inflammation and improve the function of endothelial cells by reducing the serum level of TNF-α and preventing the progression of PAH and heart diseases (93). Due to the presence of lung fibrosis, the possibility of being infected with COVID-19 is high in these patients (94). In a case report, a 70-year-old patient with COVID-19 was investigated who developed diffuse cutaneous SSc 2 weeks after receiving the first dose of ChAdOx1 nCOV-19 vaccine. The patient had no history of contact with toxic substances. In the tests performed, ANA was positive and ENA was negative (95).

### Vessel vasculitis

In the case report, a 71-year-old woman who was suffering from EGPA was treated with medicine for 7 years and has recovered to a great extent. The patient received the first dose of BNT162b2 mRNA vaccine 3 months after recovery (96). Ten days after receiving the vaccine, the patient was referred to the medical center complaining of increased myalgia and polyarthralgia, which appeared immediately after receiving the vaccine (96). The patient's clinical symptoms and conditions worsened continuously during hospitalization, but the RT-PCR is related to his COVID-19. It was always negative. CT scan of the patient had a magnifying glass view (96). Also, laboratory tests such as CRP, ESR, p-ANCA and Birmingham Vasculitis Activity Scores (BVAS) were above the normal range. The patient was treated with corticosteroids and was discharged after 12 days of hospitalization. First, she was treated with intravenous pulse methylprednisolone for 3 days and then was treated with oral prednisone for 6 months (96).

In another report, it is stated that a 46-year-old man went to the hospital a week after receiving the second dose of Pfizer BioNTech COVID-19 vaccine, complaining of severe abdominal pain since 6 days ago (97). The first day after receiving the vaccine, the patient had fever and seizures. The patient's CRP level was slightly higher than normal (97). Serological panels for autoimmune and vasculitis were negative But in the CT scan, inflammation indicating focal vasculitis was observed, and MRA confirmed these observations. The patient was treated and discharged from the hospital after 1 week, but was still under follow-up for 9 months. The patient was treated with steroids in the first 6 months of follow-up and azathioprine 150 mg daily in the next 3 months and completely recovered (97).

## Renal complications

Kidneys can be widely affected by SARS-CoV-2 due to the high expression of ACE2, which is expressed more in proximal convoluted tubules (PCTs). Therefore, the most common type of injury following COVID-19 infection is tubular injury. Other reported clinical pictures include nephrotic syndrome and glomerular injury (e.g., minimal change disease) (98, 99). Several case reports of acute kidney injuries in patients with or without prior renal pathology following vaccination (100–102). However, it has been reported that vaccination could exert a protective effect against acute kidney injury. While the infection was associated with an increased risk of acute kidney injury (125.4 events per 100,000 persons), vaccination with BNT162b2 mRNA vaccine could reduce the risk (−4.6 events per 100,000 persons) (103). The pathophysiology of SARS-CoV-2-related renal injury is presumed to be multifactorial through direct (i.e., virus or vaccine components) or indirect (i.e., immune-mediated like cytokine storm or hyperactivity of T cells) effects (104). After vaccination, suspension to kidney injury encompasses manifestations like oliguria or anuria, edema or anasarca, hypertension, and dyspnea due to pleural effusion (98). Laboratory data confirming the diagnosis include a variety of renal-associated factors' impairment due to underlying pathology. For instance, minimal change disease exhibits proteinuria, normal to increased creatinine, and podocyte injury in a light microscopy assay (101). Acute tubular necrosis can be presented with increased creatinine and urea nitrogen, proteinuria, hypoalbuminemia, biopsy findings of diffuse PCT injury, lymphocyte infiltration, and cell necrosis (98, 99).

Furthermore, hypodensity of renal parenchyma may have been seen in computed tomography (CT) (104). Treatment consists of two important approaches; firstly, kidney protection from further injury by adjusting input and output fluids, excluding nephrotoxic drugs, and monitoring creatinine levels. Secondly, early immunosuppressive therapy (non-selective or selective) or RRT due to the patient condition (104, 105).

## Dermatologic complications

### Erythema multiforme

Erythema multiforme (EM), an inflammatory dermatologic disorder, is linked chiefly to infections (most commonly: herpes simplex and *Mycoplasma pneumoniae*), although various triggers, such as many other infectious agents, immunizations, medications, and even various diseases, have also been identified. Acral, targetoid papules, consisting of three distinct concentric zones, are the hallmark lesions of this disease. It is necessary to emphasize that vaccine-induced EM has been known for a long time, with 984 cases reported to the Vaccine Adverse Event Reporting System (VAERS) (106). Furthermore, both as typical acral lesions in younger individuals and more widespread, atypical lesions in adults, EM-like reactions have already been linked to SARS-CoV-2 infection (107). The structure codified in mRNA COVID-19 vaccines, SARS-CoV-2 spike protein, was shown immunohistochemically in the epithelium and endothelial cells in the eccrine ducts in those individuals. EM is a rare adverse effect of many other vaccines, and recent studies link this reaction to mRNA vaccines (108). It has been suggested that a T-cell trigger by viral antigen-positive cells containing the HSV-DNA polymerase gene plays a vital role in EM pathogenesis, and it causes viral gene expression in the recruitment and skin (109). EM's clinical manifestations are diverse, and they can also manifest as atypical palpable lesions with erythematous dusky bodies surrounded by a paler halo. To rule out inflammatory, autoimmune, or malignant disorders, swabs are performed for HSV-PCR, Tzanck smear, or other serological tests. Direct and indirect immunofluorescence may help differentiate EM and distinguish it from other lesions of bullous vesicles. EM is managed with symptomatic treatments. The lesions may heal in 3 to 6 weeks, but patients with severe EM may need to be hospitalized for antiviral therapy, hydration, analgesics, and systemic steroids (106, 109).

### Chilblains

Repeated exposure to cold air may cause inflammation in small blood vessels, called chilblains, causing swelling, blistering, itching, and red patches on hands and feet (110). The exact pathophysiology remains unclear since the chilblain-like lesions due to COVID-19 are common features with idiopathic and autoimmune-related chilblains. COVID-19 has various clinical manifestations, including pernio/chilblains-like lesions, a condition termed “COVID-19 toes.” However, after receiving the vaccines, this condition might be associated with COVID-19 (111). These lesions have been reported post-Pfizer and -CoronaVac (inactivated vaccine) vaccinations (112, 113). These lesions could be extremely painful and last for up to 150 days after vaccination (114, 115). Anticoagulant therapy (apixaban) and low-dose aspirin were prescribed for the patient until circulating immune complexes were obtained after 14 days (114).

## Hematologic complications

### Disseminated intravascular coagulation

Disseminated intravascular coagulation) DIC) leads to extensive fibrin deposition with the formation of extensive microvascular thrombosis (116). During coagulation, coagulation factors decrease, and platelets accumulate, thereby reducing clotting protein (116). Infection by the SARS-CoV-2 increases the risk for systematic multi-organ complications and venous and arterial thromboembolism (116). CT scan demonstrated multiple subacute intra-axial hemorrhages in atypical locations, such as the right frontal and temporal lobes. A successive CT angiography of the chest added the findings of multiple contrast-filling defects with multi-vessel involvement: at the level of the left interlobar artery, of the right middle lobe segmental branches of the left upper lobe segmental branches, and the right interlobar artery (116). A plain old balloon angioplasty (POBA) of the right coronary artery was conducted, with the restoration of distal flow but with the persistence of extensive thrombosis of the vessel (116). An abdomen CT angiography demonstrated filling defects at the right supra-hepatic vein level and the left portal branch level. Bilaterally, it was adrenal hemorrhage and blood in the pelvis (116). An MRI on the same day demonstrated the presence of an acute basilar thrombosis associated with the superior sagittal sinus thrombosis. Alternative HIT-compatible anticoagulants prescribe in case of acute thrombocytopenia/thrombosis (116).

### Deep vein thrombosis and pulmonary thromboembolism

Deep venous thrombosis (DVT) and pulmonary thromboembolism (PTE) exist on the spectrum of venous thromboembolic disease (VTE) (117). DVT is known as the formation of blood clots (thrombi) in the deep veins. It usually affects the deep leg veins (e.g., the calf veins, popliteal vein, or femoral vein) or the deep veins of the pelvis. DVT is a potentially dangerous condition, leading to preventable morbidity and mortality (118). PE occurs when a thrombus migrates from the venous circulation to the pulmonary vasculature, lodging in the pulmonary arterial system. The clinical manifestation of acute PE ranges from incidentally discovered and asymptomatic to massive PE, leading to death (117). Post-vaccination DVT and PTE have been reported in a few cases worldwide. Infection could significantly increase the risk of DVT (RR of 3.78; 43.0 events per 100,000 persons) and PE (RR of 12.14; 61.7 events per 100,000 persons). Nonetheless, the BNT162b2 mRNA vaccine was not associated with an increased risk for these adverse effects (RR of 0.87; −1.1 events per 100,000 persons and RR of 0.56; −1.5 events per 100,000 persons for DVT and PE, respectively) (103). Duplex ultrasonography of the lower limbs demonstrated acute DVT involving the superficial femoral, common femoral, popliteal, anterior tibial, posterior tibial, and deep calf veins (119). The patient underwent computed pulmonary tomography angiography (CTPA) due to tachycardia, which demonstrated saddle thrombus in the bifurcation of the pulmonary trunk and 40 extensive bilateral main pulmonary arteries emboli extending to both lobar segmental and sub-segmental branches (119).

Sporadic cases report viral vector vaccine injection, vaccine-induced immune thrombotic thrombocytopenia (VITT), and cerebral venous sinus thrombosis (CVST). Generally, CVST occurs in young adults, particularly young women. In most cases, a risk factor is identified in patients (120–123). With disease progression, focal neurological deficits may develop due to seizure and venous infarction, more commonly observed in patients with CVST than the other stroke subtypes. Full recovery is achievable with timely disease diagnosis and treatment (124). The SARS-CoV-2 infection has also been proved to lead to CVST development in several studies (124, 125). SARS-CoV-2 VITT is a novel phenomenon that might occur in post-viral vector COVID-19 vaccines.

Contrary to the previous reports of post-vaccination thrombotic thrombocytopenia, CVST is reported in these patients after the COVID-19 vaccination. Clinically, VITT mimics spontaneous autoimmune heparin-induced thrombocytopenia (HIT). HIT occurs due to the complexion of heparin with platelet factor 4 (PF4) platelet-activating IgG antibodies. Next, the mentioned complex binds to the FcRγIIA receptors in platelets, activates platelets, and forms platelet microparticles (126). After that, microparticles start to form blood clots inducing the prothrombotic cascade, leading to platelet depletion and thrombocytopenia. Also, the reticuloendothelial system, especially the spleen, aggregates thrombocytopenia through antibody-coated platelet removal (126–129). Vaccine interaction with PF4 is considered a potential role in VITT pathogenesis.

This phenomenon may be attributed to the possible binding of vaccine-free DNA to PF4, which may trigger the PF4-reactive autoantibodies in the setting of VITT (130). (1) Moderate to severe thrombocytopenia. Note that mild thrombocytopenia may be observed in some cases, especially in the initial stages of VITT, (2) Thrombosis often occurs in the CVST forms (patients may have a headache) or splanchnic veins thrombosis [patients may have back or abdominal pain (or both), in addition to nausea and vomiting]. Less commonly, arterial thrombosis may occur, and (3) ELISA confirms positive PF4 “HIT” (heparin-induced thrombocytopenia) (131). Temporary headaches are among the common side effect of vaccination, though persistent headache, petechiae, blurred vision, easy bruising, or bleeding suggests considering CVST after VITT (131). Subarachnoid hemorrhage (SAH) and intracerebral hemorrhage (ICH) were observed in nearly half of the patients. Patients' platelet count ranged between 5,000 and 127,000/μL, and D-dimer and PF4 IgG Assay were positive in most cases. Of 49 CVST patients, a minimum of 19 patients died (39%) due to CVST and VITT complications (132). Heparin should not be administered in suspected cases until ruled out VITT (131). Close teamwork among hematologists, vascular neurologists, and other relevant consultants is the cornerstone of CVST and VITT-associated systemic thrombosis management (124). Despite the limited data regarding treatment strategies, daily IVIG administration (1 g/kg body weight) for 2 days is recommended following sending PF4 antibodies (124). IVIG hinders antibody-mediated platelet clearance; also, it may block FcRγIIa receptors of platelets and thus lead to downregulation of platelet activation (127).

Moreover, some experts have suggested administering high-dose glucocorticoids, which enhance the platelet count within days (127). On the other hand, plasmapheresis may be considered a potential therapeutic approach since it may temporarily remove pathologic antibodies and correct the coagulopathy (133). Platelet transfusion is contraindicated since it may lead to additional antibody-mediated platelet activation and coagulopathy (133). Non-heparin anticoagulants, such as direct thrombin inhibitors (bivalirudin, argatroban), indirect (antithrombin-dependent) factor Xa inhibitors (fondaparinux, danaparoid), and direct oral factor Xa inhibitors (rivaroxaban, apixaban), at their therapeutic anticoagulant dosage may be considered (130). In patients with severe thrombocytopenia (i.e., < 20,000/μL) or patients with reduced fibrinogen levels, alteration of dosing strategy is mandatory (124). Parenteral drugs with a short half-life are preferred in critically ill patients (124, 130). In patients with secondary ICH, anticoagulation is obligatory in CVST for progressive thrombosis prevention (124). In patients with full platelet count recovery, with no other contraindications, it is recommended to use vitamin K antagonists or direct oral anticoagulants for chronic/subacute management (124).

### Splanchnic vein thrombosis

Splanchnic vein thrombosis (SVT), including mesenteric, portal, splenic vein thrombosis, and the Budd-Chiari syndrome, manifests venous thromboembolism in an unusual site. Portal vein thrombosis and Budd-Chiari syndrome are the most and the least common presentations of SVT, respectively. In February 2021, a considerable number of VTE in unusual sites (CVST and SVT) in combination with thrombocytopenia were observed in individuals receiving the COVID-19 vaccine. This issue prompted the temporary suspension of the administration of such vaccination by the EMA on March 15, 2021, in several countries, including Austria, Germany, the United Kingdom, France, and Norway (134, 135). Other cases of SVT have also been reported after the COVID-19 vaccination (130). All patients manifested concomitant thrombocytopenia (median nadir of a platelet count of 20,000/μL; range between 9,000 and 107,000/μL), and none of the patients had previously received any form of heparin earlier than the onset of symptoms. Diagnostic evaluation is usually affected by the lack of specificity of clinical manifestations: the presence of one or more risk factors in a patient with a high clinical suspicion could indicate—the execution of diagnostic tests. Doppler ultrasonography is the first-line diagnostic tool since its accurate and has wide availability.

Further assessments, such as magnetic resonance angiography and computed tomography, should be executed in cases with suspected SVT-related complications, suspected thrombosis of the mesenteric veins, or complete information after Doppler ultrasonography (136). Symptom onset started between 4 and 16 days post-vaccination. The same treatment as VITT (mentioned earlier) was also suggested for SVT (130).

## Lymphadenopathy

Besides the abundant reports of prominent lymphadenopathy, there have been several cases of silent lymphadenopathy following COVID-19 vaccination in women undergoing imaging for breast cancer screening (137–141). Reactive lymphadenopathy has been reported following all the currently available COVID-19 vaccines, assumed as a common side effect of vaccination (142). Barda et al. reported that BNT162b2 vaccination increased the risk of lymphadenopathy (RR of 2.43; 78.4 events per 100,000 persons). However, infection was not associated with a meaningful risk of lymphadenopathy (103). It is believed that post-vaccination lymphadenopathy appears more common after mRNA vaccines (0.3% in BNT162b2 and 1.1% in Moderna vaccine) (143–145). However, Lymph node enlargement has not conclusively resulted from the COVID-19 vaccines. This phenomenon has previously been reported following Bacillus Calmette-Guerin (BCG), smallpox, human papillomavirus (HPV), H1N1 influenza A virus, and anthrax vaccines (142, 146–151). However, none of these vaccines have been administered massively as SARS-CoV-2 vaccines, and clinical experience suggests a notably higher incidence of lymphadenopathy following COVID-19 vaccines than other vaccines. Lymphadenitis and lymphadenopathy associated with COVID-19 vaccination usually occur within 4 weeks of administration and have been reported in almost all body parts, including axillary, pectoral, supraclavicular, cervical, inguinal, and even intraparotid regions (141, 143, 152, 153). The axillary region seems to be the most common location for vaccine-associated lymph node enlargement. However, the increasing rate of supraclavicular lymphadenopathy following vaccination indicates that vaccines are injected at a higher location than recommended (154). This complication has been detected through ultrasound, PET/CT imaging, or MRI in post-COVID-19 vaccinated individuals (137). Moreover, the use of PET/CT imaging in the study of hypermetabolic lymphadenopathy following COVID-19 vaccination is growing, and there are a considerable number of studies have utilized this method (155–159). The delicate features of 18F-FDG PET/CT findings in these studies could prevent inessential biopsy and certify the accuracy of staging and restaging (160).

Nonetheless, we should not underestimate the significance of evaluation for malignant causes of lymphadenopathy in vaccinated individuals since vaccination is a well-known but uncommon cause of lymphadenopathy. Fine needle aspiration is the best method for excluding cancer and metastasis (161). Nevertheless, unnecessary biopsies of benign reactive lymph nodes should be avoided (162). Therefore, there should be a protocol for evaluation. Some authorities believe that if lymphadenopathy appears within 6 weeks of vaccination in a patient with no history of malignancy, the problem is ipsilateral to the vaccine injection site. It is supposed to be vaccine-related; otherwise, assessment for other causes, particularly neoplasms, should be done (163, 164). In individuals with pre-existing unilateral cancer, vaccination should be given contralaterally if possible to avoid misinterpretation (165). According to new reports, COVID-19 vaccines could have an association with the relapse of pre-existing lymphoma. For instance, Brumfiel et al. presented a case of primary cutaneous anaplastic large-cell lymphoma who experienced the recurrence of the previous disease after 2-day-ago vaccination with the first dose of BNT162b2 vaccine, while he had demonstrated no relapse after receiving radiotherapy in the last months (166).

Furthermore, Panou et al. (167) reported two patients with cutaneous T-cell lymphoma relapse after Oxford-AstraZeneca COVID-19 vaccination who had been in the remission phase for several years. In the first case, a 60-year-old man with previous folliculotropic mycosis fungoides (stage T1aN0M0 for 2 years) developed a CD30^+^ large cell transformation on the circumferential area of the previous patch just 1 week following the second dose of Oxford-AstraZeneca COVID-19 vaccine. The second, a 73-year-old woman with stage T1a/IA mycosis fungoides and type A lymphomatoid papulosis had been in remission for 7 years. However, she demonstrated a lesion at the site of previous lymphomatoid papulosis only 10 days following the first vaccine injection. Then, the histological tests revealed the reappearance of the previous type A lymphomatoid papulosis (167). Although the underlying mechanism of lymphoma trigger caused by COVID-19 vaccination is not entirely understood, there are a few noteworthy answers to this phenomenon. For instance, there are T follicular helper (Tfh) cells that are needed for both maintenance and normal development of germinal centers (168) and also play an essential role in some malignancies and autoimmunities (169). Since a study performed by Pardi et al. (168) revealed that lipid-encapsulated nucleoside modified vaccines could trigger a robust Tfh cells hyperactivation, it can be possible to link the lymphoma relapse in the cases mentioned above with the Tfh cells hyperactivation caused by mRNA-based COVID-19 vaccines (168, 170). In a recent report, a case of angioimmunoblastic T cell lymphoma (AITL) developed a rapid progression after the booster dose of the BNT162b2 vaccine (171). Since malignant Tfh cells are present in AITL as the main hallmark, it is possible to account for their hyperactivation as one of the main mechanisms of pre-existing lymphoma recurrence or the beginning of a new lymphoproliferative disease.

Moreover, it has been reported that RHOAG17V and TET2 mutations, in combination together, could play a key role in AITL development *in vivo* study. These mutations can make Tfh cells very sensitive to dendritic cell stimulation that they start an excessive proliferation in a T cell receptor-independent manner (172). Interestingly, the AITL mentioned above case had both RHOAG17V and TET2 mutations in his Tfh cells, which brought about this theory that they could be stimulated by the booster injection of the BNT162b2 vaccine (171). Nonetheless, given the less existing knowledge, the idea is still controversial and needs more investigations in the future. Finally, a few new studies demonstrated that the BNT162b2 vaccine could lead to lengthened germinal center reactions, probably due to Tfh over-response, which might explain the hypermetabolic lymphadenopathy following vaccination (173, 174). Another interesting phenomenon following the COVID-19 vaccination has been Kikuchi's disease (KD), histiocytic necrotizing lymphadenitis, which presents with cervical lymphadenopathy or fever of unknown origin (175). The etiology of this disease is not yet determined; however, pathogens such as Epstein-Barr virus, cytomegalovirus, varicella-zoster virus, human immunodeficiency virus, *Yersinia enterocolitica*, and *Toxoplasma gondii*, and autoimmune disorders such as systemic lupus erythematosus (SLE), antiphospholipid antibody syndrome, and scleroderma have been attributed to this condition (176). KD has been rare reports following human papillomavirus and influenza vaccines (177, 178). The diagnosis of this condition is confirmed and differentiated from malignancies by histopathology and the presence of necrosis without granulocytic cells.

## Ocular complications

### AAION and AZOOR

Various studies reported vaccine-induced ophthalmic events previously. Arteritic anterior ischemic optic neuropathy (AAION) and bilateral acute zonal occult outer retinopathy (AZOOR) are described as an abrupt presentation of photopsia and scotomas due to the damage of external retinal zones has been reported as adverse events. The pathophysiology for developing AZOOR and AAION could be ascribed to the cross-reaction of neutralizing antibodies against SARS-CoV-2 spike protein or activated helper T cells after vaccination that react with proteins and antigens in large arteries, outer retinal layers, and retinal pigment epithelial cells. Presentation of these ocular manifestations after the second dose of a vaccine shot accompanied by high levels of ESR and CRP in both cases strongly supports immune system over-activity patronaging this assertion. Diagnosis of AAION was performed based on temporal artery biopsy, macular optic coherence tomography (OCT), Fluorescein angiography (FA), indocyanine green angiography (ICG), fixed and multi-luminance electroretinography (ERG), multifocal ERG as well as images of ganglion cell complex and retinal nerve fiber layer.

Similarly, OCT, fixed and ERG, multifocal ERG, FA, ICG, and fundus autofluorescence (FAF) were applied to diagnose AZOOR. Corticosteroid pulse and oral prednisolone followed by Tocilizumab were administrated in the case of AAION. AZOOR was also treated with an intravitreal implant of dexamethasone (179).

### Acute macular neuro-retinopathy

Acute macular neuro-retinopathy (AMNR) is a rare condition with the sudden presentation of one or more paracentral scotomas causing either temporary or permanent visual impairment. The pathophysiology underlying AMNR development has not been identified yet (180). However, a few cases of AMNR have been reported following the first shot of the COVID-19 vaccination and in the cases of COVID-19 itself (181, 182). However, since it is very uncommon, establishing a cause-effect relationship is possible and needs more investigation. Diagnostic evaluations, including ophthalmoscopy, OCT, swept-source optical OCT, and microperimetry, were all suggestive of AMNR in these patients. The use of oral contraceptives is associated with AMNR development which further supports COVID-19 vaccine-induced AMNR as one of the cases was consuming OCP (183, 184).

### Paracentral acute middle maculopathy

Paracentral acute middle maculopathy (PAMM) is described as the presence of a hyper-reflective band at the level of the inner nuclear layer visualized in OCT, which indicates infarction of the inner nuclear layer. Impaired perfusion of the retinal capillary system can be associated with several causes, including occlusion of the central retinal vein, retinal artery occlusion, and the non-proliferative diabetic form of diabetic retinopathy causing inner nuclear layer infarction (185). A recent study reported a case of PAMM and giant cell arteritis-like vasculitis following COVID-19 infection. Thus, the association with COVID-19 might be possible (186). PAMM has also been reported after the Sinopharm vaccine. The mentioned case developed uncontrollable hypertension 20 min after the vaccine shot, accompanied by the simultaneous development of left eye inferior scotoma and headache. Visual acuity was decreased on admission. OCT angiography and fundus examination were all indicative of PAMM (187).

### Central serous retinopathy/chorioretinopathy

Central serous retinopathy (CSR), a common ocular disease, is described as retinal pigment epithelium (RPE) decompensation, leading to the detachment of either the neurosensory retina or the serous pigment epithelium. Symptoms of CSR include blurred vision, metamorphopsia, micropsia, dyschromatopsia, or even asymptomatic. Although CRS's pathophysiology has not been completely understood, increased permeability and thickness of choroid due to ischemia, inflammation, or hydrostatic forces have been proposed as the possible mechanism (188). mRNA vaccines induced CSR have been postulated to develop due to the presence of polyethylene glycol used in vaccine formulation, causing anaphylaxis, choroid vessel thickening, and neovascularization. The possible release of endogenous cortisol triggered by mRNA vaccines is also hypothesized to be associated with CSR development after vaccination, as high cortisol levels in serum are associated with CSR. Another probable mechanism for post-vaccination CSR is extracellular RNA presence which induces increased endothelial cell permeability and thrombus formation, which is also compatible with lobular ischemia seen in CSR. CSR has been previously reported, followed by smallpox, yellow fever, influenza, and anthrax vaccine. CSR development followed by the Pfizer vaccine was reported 69 h after the injection. OCT, OCT angiography, and FA were all suggestive of CSR. Spironolactone 50 mg daily was prescribed, and the patient became asymptomatic with routine visual tests after 3 months (189).

### Bilateral retinal detachment

Retinal detachment is an emergency medical condition that requires prompt treatment, leading to permanent blindness. There are three types of retinal detachments, including tractional and exudative, which are non-rhegmatogenous, and rhegmatogenous retinal detachment (RRD), which is the most common point-of-care ultrasound (POCUS) of the eye was suggestive for non-posterior vitreous detachment that is a form of RRD which round holes that are associated with local thinning or atrophy of retina including lattice degeneration. The patient then undergoes bilateral vitrectomies (190).

### Uveitis

Uveitis is a threatening, inflammatory eye disorder considered an ophthalmic emergency. Uveitis develops primarily due to autoimmune reactions, ocular trauma, infection, or it may be isolated (191). Uveitis development following vaccination can present a wide range of ocular manifestations such as redness of the eye, blurred vision, floaters, and sensitivity to light. Conjunctival hyperemia and eye pain can also be the clinical manifestations of vaccine-associated uveitis (191). Vaccine-associated uveitis has been previously reported following almost all the vaccines currently employed, such as the hepatitis B vaccine, the commonest vaccine-related uveitis, human papillomavirus, and influenza vaccine (192). It was found that COVID-19 vaccination was not associated with an increased risk for uveitis (RR of 1.27; 1.0 events per 100,000 persons) (103).

The pathophysiology underlying the development of vaccine-associated uveitis could be attributed to autoimmune mechanisms caused by the vaccine. The possible mechanisms involved in this autoimmunity include molecular mimicry due to the resemblance of uveal self-peptides and vaccine peptides, cytokine production, new antigen induction, surface antigen modification, B cell polyclonal activation, and adjuvant-induced inflammatory destruction (193).

Most cases of vaccine-associated uveitis are anterior, transient, not severe, and respond to topical steroids promptly. However, there have been reports of posterior uveitis and pan-uveitis, including Vogt-Koyanagi-Harada (VKH) and acute posterior multifocal placoid pigment epitheliopathy (AMPPE) in severe cases following vaccination. Previous studies showed conjunctival hyperemia, photophobia, decreased visual acuity, and eye pain. Laboratory data, including WBC count, CRP, and ESR levels, were normal with negative ANA and rheumatoid factor (RF). Slit-lamp examination and OCT results were suggestive of uveitis. Dexamethasone eye drops six times a day and atropine 1% (cycloplegic agent) twice daily were prescribed for a patient. Several diffuse scleral hyperemia lesions were observed on slit photos. Scleritis resolved 1 week after prescribing topical steroids for the patient (187). Pan uveitis-associated COVID-19 vaccine has also been reported with substantial vision loss, ocular pain, and light sensitivity. Fluorescein angiography, OCT imaging, and B-scan were used to diagnose OCT and B-scan showing choroidal thickening. The patient was prescribed oral prednisolone (50 mg/kg) and Difluprednate eye drop (192).

Vogt-Koyanagi-Harada syndrome is a rare granulomatous inflammatory disorder that targets pigmented structures, including the inner ear, eye, meninges, hair, and skin. The disease causes non-necrotizing panuveitis and exudative retinal detachment. The pathophysiology underlying VKH has been mediated by Th1 lymphocytes against melanocyte antigenic components. A case of VHK has been reported for 4 days, followed by the COVID-19 vaccine with bilateral acute vision loss. Slit photo, OCT, and Fundus examination helped diagnose VKH. Oral systemic prednisolone (1.5 mg/kg) was prescribed for the patient daily (194).

## Gastrointestinal complications

### Autoimmune hepatitis

Autoimmune hepatitis is characterized by inflammatory liver disease, which can be triggered by various factors such as viruses, bacteria, drugs, and some substances in genetically predisposed patients. Acute autoimmune hepatitis has been reported to develop, followed by hepatotropic viruses, such as hepatitis A, B, and C viruses, and non-hepatotropic viruses, including Epstein-Barr virus (EBV) (195). Recently infection with SARS-CoV-2 has been associated with autoimmune hepatitis development. Furthermore, autoimmune hepatitis happens following COVID-19 mRNA vaccines (196, 197); however, like most of the adverse events discussed in this paper, it is very infrequent, and establishing a cause-effect relationship needs more investigation. As autoimmune conditions leading to tissue destruction following severe SARS-CoV-2 infection have been reported, it could be similarly stated that molecular mimicry is responsible for the development of autoimmune hepatitis in these cases (196). The cases were negative for viral hepatitis (hepatitis A, B, C, and E, cytomegalovirus, EBV, herpes simplex virus, and HIV). Laboratory data showed elevated bilirubin, albumin, and liver enzymes, suggestive of hepatocellular injury. Double-stranded DNA antibodies (dsDNA) and antinuclear antibodies (ANA) were positive in these cases with elevated IgG levels. There was no evidence of biliary lithiasis or dilation. Histopathological evaluations were also compatible with autoimmune hepatitis, showing portal inflammation, interface hepatitis, rosette formation, and eosinophils, which increase the possibility of drug-induced autoimmune hepatitis in mentioned cases. Budesonide or prednisolone 20 mg daily can be administrated to treat COVID-19 vaccine induced autoimmune hepatitis.

## Cardiovascular complications

### Myocardial infarction

Myocardial infarction (MI) is a term used for an event of a heart attack due to the formation of plaques in the arteries' interior walls, resulting in reduced blood flow to the heart and injuring heart muscles because of a lack of oxygen supply (198). MI is a rare complication observed following the COVID-19 vaccine, but it is a significant problem and can be a life-threatening adverse event. Although infection increases the risk of MI in patients (RR of 4.47; 25.1 events per 100,000 persons), BNT162b2 vaccine was not associated with an increased risk of MI (RR of 1.07; 0.8 events per 100,000 persons) (103).

There are some potential explanations for myocardial infarction after the COVID-19 vaccine. First, prothrombotic immune thrombocytopenia induced by the vaccine has similarities to heparin-induced thrombocytopenia leading to thrombotic manifestation. Second, COVID-19 vaccines increase demand for the heart as a contributing factor, then cause a demand-supply mismatch. Third, this can result from Kounis syndrome, defined as an acute coronary syndrome caused by an allergic reaction or a strong immune reaction to various substances, including excipients, drugs, or other substances. MI clinical manifestations include chest pain which travels from left arm to neck, shortness of breath, sweating, nausea, vomiting, abnormal heart beating, anxiety, fatigue, weakness, stress, and depression (199, 200). Paraclinic findings would be Non-ST-elevation and ST-elevation with or without T segment inversion and even reciprocal changes in ECG, abnormal motion of the wall in echocardiography, high-level biomarkers such as Creatine-Kinase-MB isoform and Cardiac Troponin (the biomarker of choice), cardiac troponin I is the gold standard of MI diagnosis, and angiography to localized blot clots formation in coronary vessels (201). The aim of myocardial infarction management is thrombolysis and reperfusion of the myocardium, although a variety of drugs such as anti-platelets (aspirin), heparin, anti-anginal (β-blockers, and nitrates) might also be considered. Percutaneous coronary intervention (PCI) is for reperfusion of the myocardium. If there is no emergency percutaneous coronary intervention facility, thrombolytic therapy with 1.5 million IU/h intravenous streptokinases can be administered (199).

### Myocarditis and pericarditis

Pericarditis is inflammation of the pericardium, a two-thin-layer sac-like structure that surrounds the heart, and also myocarditis is inflammation of the myocardium (heart muscle). This inflammation can result from an immune response to an infection or other substances (202). Viral infections, such as adenovirus, coxsackievirus, herpes virus, influenza, and even SARS-CoV-2, are the most common cause of myocarditis and pericarditis (203). Previously, myocarditis and pericarditis have been reported after smallpox vaccination and less after other live viral vaccines (including measles-mumps-rubella, varicella, oral polio, or yellow fever vaccine). Recently myocarditis and pericarditis have been reported to associate with COVID-19 vaccination, especially mRNA vaccines (204). Vaccination with BNT162b2 was associated with a significantly increased risk of myocarditis compared to unvaccinated cases (RR of 3.24; 2.7 events per 100,000 persons). The infection was also associated with an increased risk of myocarditis (RR of 18.28; 11.0 events per 100,000 persons). Similarly, the infection significantly increases the risk of pericarditis (RR of 5.39; 10.9 events per 100,000 persons). However, it has been found that vaccination was not associated with an increased risk of pericarditis (1.0 events per 100,000 persons) (103). For this reason, FDA attached a caution about the risk of myopericarditis to the information sheet of mRNA anti-SARS-CoV-2 vaccines (205). Immunopathological mechanisms of COVID-19 vaccination can be theoretical risks of myocarditis and pericarditis post-COVID-19 vaccination (206). Potential hypothesized mechanisms include (1) very high antibody generation response, similar to the multisystem inflammatory syndrome in children (MIS-C) associated with SARS-CoV-2 infection, (2) anti-idiotype cross-reactive antibody-mediated cytokine expression induction in the myocardium, (3) non-specific innate inflammatory response or a molecular mimicry mechanism between the viral spike protein and an unknown cardiac protein, and (4) immunogen potential of RNA itself in vaccine and adjuvant effect production by cytokine activation of pre-existing autoreactive immune cells (205).

Although it was challenging to separate myocarditis from pericarditis in the published cases, the most common signs and symptoms are not effort-related chest pain but positional and worsened by deep breathing, which may be followed by fever, and less common are dyspnea, cough, and headache. Furthermore, symptoms onset occurred between 1 and 7 days after vaccination (205, 206). Diagnosis is based on medical history and physical examination, echocardiography, ECG findings, and blood test. Cardiac MRI and biopsy are confirmation diagnostic evaluations, but these are not available in most centers. Therefore, abnormal Lab findings, including troponin, brain natriuretic peptide, erythrocyte sedimentation rate, CRP, and cardiac antibodies, when coupled with a concerning clinical presentation and ECG, can be used to make a presumptive diagnosis (207). The most common changes in a post-COVID-19 vaccination patient's ECG are diffuse ST elevation and ST depression without reciprocal changes, T-inversion, and sinus tachycardia associated with non-specific ST/T-wave changes. Trans-thoracic echocardiography (TTE) and CMR are used to diagnose effusion and pericardial thickening (202, 207). Due to inflammation and high troponin, CRP may become high because of muscle damage resulting from myocarditis (205).

The first step evaluation should be ECG and laboratory tests such as CBC, electrolytes, renal and liver function test, CRP and troponin level, and SARS-CoV-2 reverse transcriptase-polymerase chain reaction (RT-PCR) test. Notice that normal ECG presentation and Normal troponin level do not rule out isolated pericarditis. Cardiac MRI should be performed if the clinical findings are highly probable and the cardiac troponin level is elevated (204). There is insufficient evidence supporting anti-inflammatory drug prescription for all patients post COVID-19 vaccination myocarditis or pericarditis. Generally, based on the evidence we have, pain management and NSAIDs with or without colchicine can be used for mild or moderate. Also, in severe cases, IVIG and corticosteroids might be considered. In unstable hemodynamic patients, inotrope drug and cardiogenic shock management might be required (204, 205, 207).

## Neurologic complications

### Guillain-Barré syndrome

Guillain-Barré syndrome (GBS) is a rare acute severe acquired immunomediated inflammatory polyradiculoneuropathy that affects peripheral nerves (208). The exact pathophysiology is not fully understood, but it often occurs after a recent infection (209). *Campylobacter jejuni*, CMV, HEV, Epstein–Barr virus, influenza, mycoplasma pneumoniae, and Zika virus are the most common infection associated with GBS (210). Also recently, GBS after the COVID-19 infection has been reported, but on the other hand, we also have some cases of GBS observed following COVID-19 vaccination (210, 211). Since the COVID-19 vaccines cause immunization against SARS-CoV-2 infection spike proteins, which bind to gangliosides and glycoproteins on cell surfaces, the causal connection could be the cross-reaction between antibodies produced by COVID-19 vaccines and GBS (212). Progressive, ascending, symmetrical flaccid paralysis of the limbs, simultaneously with hypo or areflexia, is the typical clinical pattern of the GBS (213) and even may include cranial nerve and respiratory muscle involvement. However, based on some reports about GBS-related post-COVID-19 vaccination, we have, it seems bifacial weakness may be the characteristic clinical manifestation of GBS-related post-COVID-19 vaccination (214). The diagnosis of GBS diagnostic criteria is mainly based on history and physical examination, electromyography and nerve conduction velocity (EMG/NCV) studies, and cerebrospinal fluid analysis as a confirmation diagnostic test. GBS treatment would be intravenous immunoglobulin IVIg (0.4 g/kg/day for 5 days) and plasma exchange (209, 210, 213).

### Stroke

An ischemic stroke could happen because of coagulopathy, blood clot formation, and thrombosis in the vasculature that carries blood to the brain (215). However, stroke and cerebral accidents are coagulopathy- and thrombosis-related complications of COVID-19. Some evidence of coagulopathy and cerebral vascular accident after the COVID-19 vaccination has recently been reported (216). However, there was no increased risk for cerebrovascular accidents after vaccination (RR of 0.84; −1.6 events per 100,000 persons) in the study by Barda et al. (103). The definite underlying mechanism is unknown. It may mimic heparin-induced thrombocytopenia with existing anti-PF4 but in the absence of heparin, also known as VITT (128). Clinical manifestation based on which vessel is affected would vary, but sudden unilateral weakness or numbness in the face or arm and legs, speech difficulty, hearing or sight loss in one or both eyes, dizziness, and confusion are the most common signs and symptoms. Typical laboratory findings would be platelet count < 100,000/μL with a high D-dimer level and an inappropriately low fibrinogen level (128).

Therefore, this is a rare but life-threatening adverse event that needs critical and rapid management. This phenomenon should be considered in patients with focal neurological deficits or other severe neurological disorders, with platelet counts under 100,000/μL up to 1 month after the COVID-19 vaccination. First step evaluation includes brain CT scan with additional venography and lab test like CBC, Retic counts, peripheral blood smear, PT, aPTT, fibrinogen, D-dimer test, antiphospholipid LDH level, paroxysmal nocturnal screening, and ADAMTS-13 should be done for suspected cases. Also, serum samples for anti-PF4 antibodies should be sent immediately (128). Since diagnosis and management of these critical and challenging situations will need close collaboration, hematologist and neurologist consultation is another prominent part of better management and should be prepared. For VITT management, heparin drugs in all forms (unfractionated heparin, or low-molecular-weight heparin, e.g., enoxaparin) and platelet transfusion because of exacerbation are all avoided (128). Nevertheless, non-heparin agents like direct oral anticoagulants (DOACs, fondaparinux, danaparoid, or argatroban) can be used depending on the clinical picture for anticoagulation. Also, IVIG administration is recommended (1 g/kg, which can be given in divided doses over 2 days) (128, 217).

### Bell's palsy

Acute onset peripheral mononeuropathy can cause paresis or paralysis of the facial nerve (seventh cranial nerve, IV) and is also known as Bell's palsy. Bell's palsy is the most common sudden onset mononeuropathy and has a potent predilection for women (218). Diabetes, obesity, hypertension, pregnancy and upper respiratory tract infection could be risk factors for the condition (219). Although the exact pathophysiology is unknown, this phenomenon could result from cranial nerve VII inflammation and edema caused by viral infections (219). The relationship between the intranasal influenza vaccine and Bell's palsy was shown in 2004 (219). However, we had reports about bell's palsy-related SARS-CoV-2 infection (220), but even some reports about bell's palsy occurring after the COVID-19 vaccination have been addressed recently (221). Barda et al. reported that vaccination could be associated with a mildly increased risk of Bell's palsy (3.5 events per 100,000 patients) (103). The mechanism of bell's palsy-related COVID-19 vaccine is under investigation, but there is some potential explanation hypothesis. First, the mRNA vaccines are associated with interferon type 1 and can cause transient lymphopenia about 1–3 days after administration, on the other hand, CD3 and 4 are down in the acute phase of bell's palsy. Second, Alpha interferon which is a type of interferon 1 can cause tolerance disruption of myelin sheath antigen (222). Therefore, SARS-CoV-2 vaccination should be considered as an additional reason for Bell's palsy besides other causes like idiopathic and viruses (222). The diagnosis is based on clinical presentation and no additional test. Although Bell's palsy will be cured spontaneously in many cases, a high-dose corticosteroid as a routine dosage based on guidelines would be helpful, and in severe cases, antiviral agents such as valacyclovir or acyclovir might be effective to enhance outcome (218).

### Transverse myelitis

Transverse myelitis (TM) is a rare, acquired focal neurological disorder resulting from an inflammatory condition that affects the spinal cord without any compression. Demyelinating disorders such as multiple sclerosis, neuromyelitis optica (NMO), infections, and vaccines are the most common causes (223). Although post-vaccination transverse myelitis is uncommon, some studies on TM-related vaccines after diphtheria, Tetanus, pertussis, measles, mumps, rubella, HBV, seasonal influenza, and oral polio vaccine administration have been reported. Recently, transverse myelitis following COVID-19 vaccination has been reported (224)—some reports about acute transverse myelitis following SARS-CoV-2 infection (225, 226).

The definitive mechanism of this condition is not apparent. Some supposals can justify this phenomenon, but molecular mimicry is the most common mechanism because of the similarity between microbial pathogen antigens and self-antigens (227). Clinical manifestations vary based on the place of involvement, but transverse myelitis is described by the sudden onset of acute or sub-acute bilateral sensory-motor and autonomic dysfunction with a clearly defined sensory level (227). Most patients present with legs and arms weakness, pain, tingling, burning sensation, sensory alteration, bladder dysfunction, urinary retention, defecation disturbance, paraplegia, and hyperactive reflexes (223, 227). In general, the first step in transverse myelitis diagnosis is history and physical examination. The next step would be ruling out the compressive etiologies by gadolinium-enhanced MRI and, after structural abnormality investigation, cerebrospinal fluid (CSF) analysis for inflammation and defining demyelinating extension.

Further workup may be performed to investigate other possible causes like infections or vitamin B12 deficiency (228). Currently, we do not have specified guidelines for the treatment of TM following COVID-19 vaccination (223), but treatment would be the administration of steroids (1 g intravenous methylprednisolone daily for 3–5 days). Plasmapheresis therapy can be used if the patient's symptoms do not improve (224, 229, 230).

## Conclusion

There have been abundant reports of adverse events following COVID-19 vaccines, though many of which are self-limited and non-serious. As mentioned before, most of the post-COVID-19 vaccination adverse events discussed in this study are very uncommon. Since there is no formal evidence that COVID-19 vaccination is responsible in most cases, establishing a cause-effect relationship needs more investigation. Nevertheless, some of the rare adverse events are reported to be life-threatening (Table 2). Therefore, it is vital to monitor at-risk vaccinated people for such adverse events, and if necessary, appropriate diagnostic modalities and therapeutic options should be utilized to minimize such catastrophic events. Also, as the incidence of such rare adverse events is significantly lower after administering COVID-19 vaccines than the disease itself, the benefits of vaccination outweigh its risks for all genders and age groups. Hence, all stakeholders, medical professionals, and governments should encourage people to receive the COVID-19 vaccine.

**Table 2:** Summary of the rare adverse events following COVID-19 vaccination.

**Complication**		**Indicators**	**Treatment**
Autoimmune	Acquired hemophilia A	Bruise or ecchymosis in the skin, prolonged activated partial thromboplastin time (APTT), decreased FVIII level, elevated FVIII inhibitor.	1) Homeostasis normalization, complete coagulation cascade with recombinant activated factor VII (rFVIIa) and activated prothrombin complex concentrate (APCC). 2) Corticosteroids (mostly high dose prednisone), cyclophosphamide, and rituximab (in refractory cases).
	Immune-mediated thrombocytopenia	Petechiae, purpura, bruising, and mucosal bleeding. Platelet count under 5,000/μL is life-threatening, with a high risk of intracranial hemorrhage (ICH).	IV fluids, corticosteroids, IVIG, platelet transfusion, rituximab, and thrombopoietic agents (e.g., romiplostim, eltrombopag). Corticosteroids (e.g., dexamethasone, methylprednisone) and IVIG.
	Anaphylaxis	Throat closure, upper airway swelling, nausea-vomiting, tachycardia, difficulty breathing without wheeze or stridor, angioedema, hypotension, and dry cough. Diagnosis is primarily clinical and requires immediate actions.	1) Epinephrine (0.01 mg/kg) while simultaneously assessing airway, breathing, circulation, and mental status. 2) Antihistamine and glucocorticoids
	Capillary leak syndrome	Hypoalbuminemia and increased creatinine level, lactate dehydrogenase, creatine kinase, aspartate aminotransferase.	Solnatide, FX06, and Bβ15-42, vasopressors (e.g., norepinephrine, vasopressin, and epinephrine), antibiotics, volume replacement, a high dose of corticosteroid, and IVIG (1 g/kg). Fasciotomy, and in the case of acute renal injury, renal replacement therapy may be needed. Moreover, prophylaxis with IVIG.
	IgA vasculitis and leukocytoclastic vasculitis (hypersensitivity vasculitis)	Purplish appearance, Elevated CRP, ESR, urea, creatinine, serum amyloid A levels, IgM, IgA, and anti-spike IgG. Histopathological evaluations and direct immunofluorescence analysis.	Methylprednisolone, Deflazacort, Paracetamol, and Prednisolone taper.
	Urticarial vasculitis	Elevated red rashes on the skin, which are itchy. Elevated CRP and histopathological evaluations.	Oral Indomethacin, Levocetirizine tablet, and topical calamine lotion.
	Cutaneous vasculitis	Physical examination and skin biopsy. The occurrence of a peculiar post-COVID-19 vaccination maculopapular rash.	Systemic antihistamine and local steroid therapy.
	Rheumatoid arthritis and reactive arthritis	Elevated ESR and CRP levels with abnormal ultrasound evaluation of the swollen limb and arthrocentesis.	Intra-articular steroid.
	IgA nephropathy	Urine analysis, Kidney ultrasound, evaluation of immunoglobulin A levels, and kidney biopsy.	Losartan and methylprednisolone.
	Thyroiditis	Suppressed thyroid-stimulating hormone (TSH) levels accompanied by elevated triiodothyronine (T3) and thyroxine (T4), increased levels of inflammatory markers (ESR, CRP), ultrasound findings, and negative thyroid antibodies. Pharyngitis, moderate fever, diffuse myalgia, and cervical pain that radiates to the jaw and ears.	Methylprednisolone, propranolol, and ibuprofen.
	Dermal filler reaction	Tenderness, swelling, erythema, and nodules. Observation is the first approach.	Tetracycline and macrolides For non-inflammatory nodules, hyaluronidase with or without intralesional steroids can be utilized. Low doses of ACE inhibitors.
	Systemic sclerosis	Vascular abnormalities, organ involvement, and skin fibrosis, Pulmonary fibrosis and pulmonary hypertension. Positive antinuclear antibodies (ANA).	Etanercept and infliximab.
	Vessel vasculitis	Myalgia and polyarthralgia, fever and seizures. Elevated CRP, ESR, p-ANCA.	Methylprednisolone pulse, followed by prednisone (alternative treatments, such as azathioprine could be used).
Renal		Oliguria or anuria, edema or anasarca, hypertension, dyspnea due to pleural effusion. Renal-associated factors' impairment. For instance, minimal change disease exhibits proteinuria, normal to increased creatinine, and podocyte injury in lite microscopy assay. Acute tubular necrosis with increased creatinine and urea nitrogen, proteinuria, hypoalbuminemia, biopsy findings of diffuse PCT injury, lymphocyte infiltration, cell necrosis, hypodensity of renal parenchyma may have been seen in computed tomography (CT).	1) Adjusting input and output fluids, excluding nephrotoxic drugs, and monitoring creatinine level. 2) Early immunosuppressive therapy or renal replacement therapy (RRT).
Dermatologic	Erythema multiforme	General appearance and skin biopsy. Swabs are performed for HSV-PCR, Tzanck smear, or other serological tests. Direct and indirect immunofluorescence.	Symptomatic treatments, antiviral therapy, hydration, analgesics, and systemic steroids.
	Chilblains	Pernio/chilblains-like lesions of the toes.	An anticoagulant therapy (apixaban) and low-dose aspirin
Hematologic	Disseminated intravascular coagulation	Angio-CT and MRI	alternative HIT-compatible anticoagulants
	Deep vein thrombosis and pulmonary thromboembolism	Ultrasound examination	Oral anticoagulant (rivaroxaban)
	Vaccine-induced immune thrombotic thrombocytopenia and cerebral venous thrombosis	Thrombocytopenia in the initial stages of VITT, thrombosis often occurs in the CVST forms or splanchnic vein thrombosis, arterial thrombosis, ELISA confirm positive PF4 “HIT” (heparin-induced thrombocytopenia)	(IVIG) administration, high dose glucocorticoids, Plasmapheresis, Non-heparin anticoagulants, such as direct thrombin inhibitors (Bivalirudin, Argatroban), Indirect (antithrombin-dependent) factor Xa inhibitors (Fondaparinux, Danaparoid), and direct oral factor Xa inhibitors (Rivaroxaban, Apixaban). In patients with full platelet count recovery, with no other contraindications, it is recommended to use vitamin K antagonists or direct oral anticoagulants for chronic/subacute management.
	Splanchnic vein thrombosis	Doppler ultrasonography, magnetic resonance angiography, and computed tomography.	The same treatment as VITT.
Lymphatics	Lymphadenitis	Assessment for neoplasms, histopathology, and the presence of necrosis without granulocytic cells.	Fine needle aspiration.
Ocular	AAION and AZOOR	AAION: Temporal artery biopsy, macular optic coherence tomography (OCT), Fluorescein angiography (FA), indocyanine green angiography (ICG), fixed and multi-luminance electroretinography (ERG), multifocal ERG as well as images of ganglion cell complex and retinal nerve fiber layer. AZOOR: OCT, fixed and multi-luminance electroretinography (ERG), multifocal ERG, FA, and ICG, as well as fundus autofluorescence (FAF).	AAION: Corticosteroid pulse, oral prednisolone followed by Tocilizumab. AZOOR: Intravitreal implant of dexamethasone.
	Acute macular neuro-retinopathy	Ophthalmoscopy, OCT, swept-source optical OCT, and microperimetry	Oral contraceptives.
	Central serous retinopathy/chorioretinopathy	Followed by smallpox, yellow fever, influenza, and anthrax vaccine. OCT, OCT angiography, and FA.	Spironolactone
	Bilateral Retinal Detachment	Point-of-care ultrasound (POCUS) of the eye.	Bilateral vitrectomies.
	Uveitis	Fundus examination, spectral domain-OCT, B-scan ultrasonography. Conjunctival hyperemia and eye pain.	Dexamethasone eye drops and atropine 1% (cycloplegic agent), topical steroids, oral prednisolone, and difluprednate eye drop.
Gastrointestinal	Autoimmune hepatitis	Bilirubin, albumin, and liver enzymes suggest hepatocellular injury, double-stranded DNA antibodies, antinuclear antibodies, and histopathological evaluations.	Budesonide or Prednisolone.
Cardiovascular	Myocardial infarction	Chest pain, shortness of breath, sweating, nausea, vomiting, abnormal heartbeat, anxiety, fatigue, weakness, stress, depression. Non-ST-elevation and ST-elevation, abnormal wall motion in echocardiography, high-level biomarkers, such as Creatine-Kinase-MB isoform and Cardiac Troponin.	Thrombolysis and reperfusion of the myocardium, anti-platelets (aspirin), heparin, antianginal (β-blockers, and nitrates). Percutaneous coronary intervention (PCI), intravenous streptokinase.
	Myocarditis and pericarditis	ECG and laboratory tests, including CBC, electrolytes, renal and liver function, CRP and troponin level, and SARS-CoV-2 RT-PCR.	Pain management and NSAIDs, IVIG, and corticosteroids.
Neurologic	Guillain-Barré syndrome	Physical examination, electromyography and nerve conduction studies (EMG/NCS), and cerebrospinal fluid analysis.	Intravenous immunoglobulin IVIG and plasma exchange.
	Stroke	The first step evaluation includes brain CT with additional venography and lab tests, like CBC, Retic counts, peripheral blood smear, PT, PTT, APTT, fibrinogen, D-dimer test, antiphospholipid LDH level, paroxysmal nocturnal screening, and ADAMTS-13 should be done. Also, a serum sample for the anti-PF4 antibody should be sent immediately. Since diagnosis and management of these critical and challenging situations will need close collaboration, hematologist and neurologist consultation is another important part of the appropriate management and should be undertaken.	Non-heparin agents like DOACs (i.e., fondaparinux, danaparoid, or argatroban) or IVIG administration.
	Bell's palsy	Clinical presentation, with no additional tests.	A high-dose corticosteroid or antiviral agents, such as valacyclovir or acyclovir.
	Transverse myelitis	History and physical examination, MRI, CSF analysis.	Steroids and plasmapheresis therapy.

## Author contributions

ZM: data collection and writing the manuscript. JL, AS, AA, ZV, TS, and MS helped with manuscript writing and contributed substantial revisions to the manuscript's content. MP, AB, EH, ND, and SA: data collection and helped with manuscript writing. RH: visualization, software, and helped with manuscript writing. MB: data collection, helped with manuscript writing, and contributed substantial revisions to the manuscript's content. SE: design of the research study and supervision. All authors contributed to the article and approved the submitted version.

## Conflict of interest

Author TS reports that he provides strategic and scientific recommendations as a member of the Advisory Board and speaker for Novocure, Inc. and also as a member of the Advisory Board to Galera Therapeutics, which are not in any way associated with the content or disease site as presented in this manuscript. The remaining authors declare that the research was conducted in the absence of any commercial or financial relationships that could be construed as a potential conflict of interest.

## Publisher's note

All claims expressed in this article are solely those of the authors and do not necessarily represent those of their affiliated organizations, or those of the publisher, the editors and the reviewers. Any product that may be evaluated in this article, or claim that may be made by its manufacturer, is not guaranteed or endorsed by the publisher.

## Glossary

SARS-CoV-2, severe acute respiratory syndrome coronavirus 2; COVID-19, coronavirus disease 2019; AESI, adverse events of special interest; AHA, acquired hemophilia A; FVIII, factor VIII; aPTT, activated partial thromboplastin time; rFVIIa, recombinant activated factor VII; aPCC, activated prothrombin complex concentrate; ITP, Immune-mediated thrombocytopenia; ICH, intracranial hemorrhage; AEFI, adverse events following immunization; CARPA, complement activation pseudo-allergy; PEG, polyethylene glycol; CLS, Capillary leak syndrome; PCLS, pulmonary CLS; SCLS, systemic CLS; RRT, renal replacement therapy; TSH, thyroid-stimulating hormone; T3, triiodothyronine; T4, thyroxine; HA, Hyaluronic acid; PCT, proximal convoluted tubules; ACE2, angiotensin-converting enzyme 2; CT, computed tomography; EM, Erythema multiforme; VAERS, vaccine Adverse Event Reporting System; DIC, Disseminated intravascular coagulation; POBA, plain old balloon angioplasty; DVT, Deep venous thrombosis; PTE, pulmonary thromboembolism; VTE, venous thromboembolic disease; CTPA, computed pulmonary tomography angiography; VITT, vaccine-induced immune thrombotic thrombocytopenia; CVST, cerebral venous sinus thrombosis; PF4, platelet factor 4; SAH, Subarachnoid hemorrhage; ICH, intracerebral hemorrhage; SVT, Splanchnic vein thrombosis; BCG, Bacillus Calmette-Guerin; HPV, human papillomavirus; KD, Kikuchi's disease; SLE, systemic lupus erythematosus; AAION, Arteritic anterior ischemic optic neuropathy; AZOOR, acute zonal occult outer retinopathy; OCT, optic coherence tomography; FA, Fluorescein angiography; ERG, electroretinography; FAF, fundus autofluorescence; AMNR, Acute macular neuro-retinopathy; PAMM, Paracentral acute middle maculopathy; CSR, Central serous retinopathy; RPE, retinal pigment epithelium; RRD, rhegmatogenous retinal detachment; POCUS, point-of-care ultrasound; VKH, Vogt-Koyanagi-Harada; AMPPE, acute posterior multifocal placoid pigment epitheliopathy; RF, Rheumatoid factor; EBV, Epstein-Barr virus; MI, Myocardial infarction; PCI, Percutaneous coronary intervention; MIS-C, multisystem inflammatory syndrome in children; TTE, Trans-thoracic echocardiography; RT-PCR, reverse transcriptase-polymerase chain reaction; GBS, Guillain-Barré syndrome; EMG/NCV, Electromyography and nerve conduction velocity; DOACs, direct oral anticoagulants; CD, Cluster of differentiation; TM, Transverse myelitis; NMO, neuromyelitis optica; CSF, cerebrospinal fluid.
